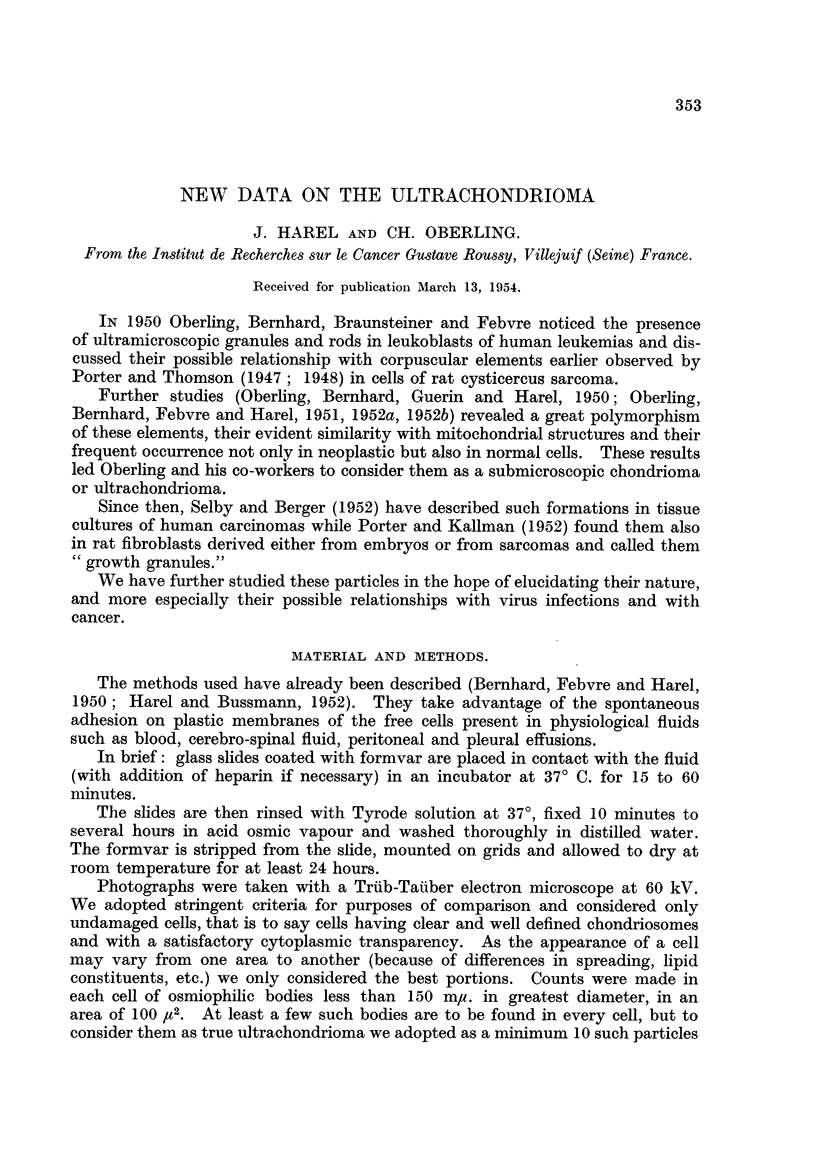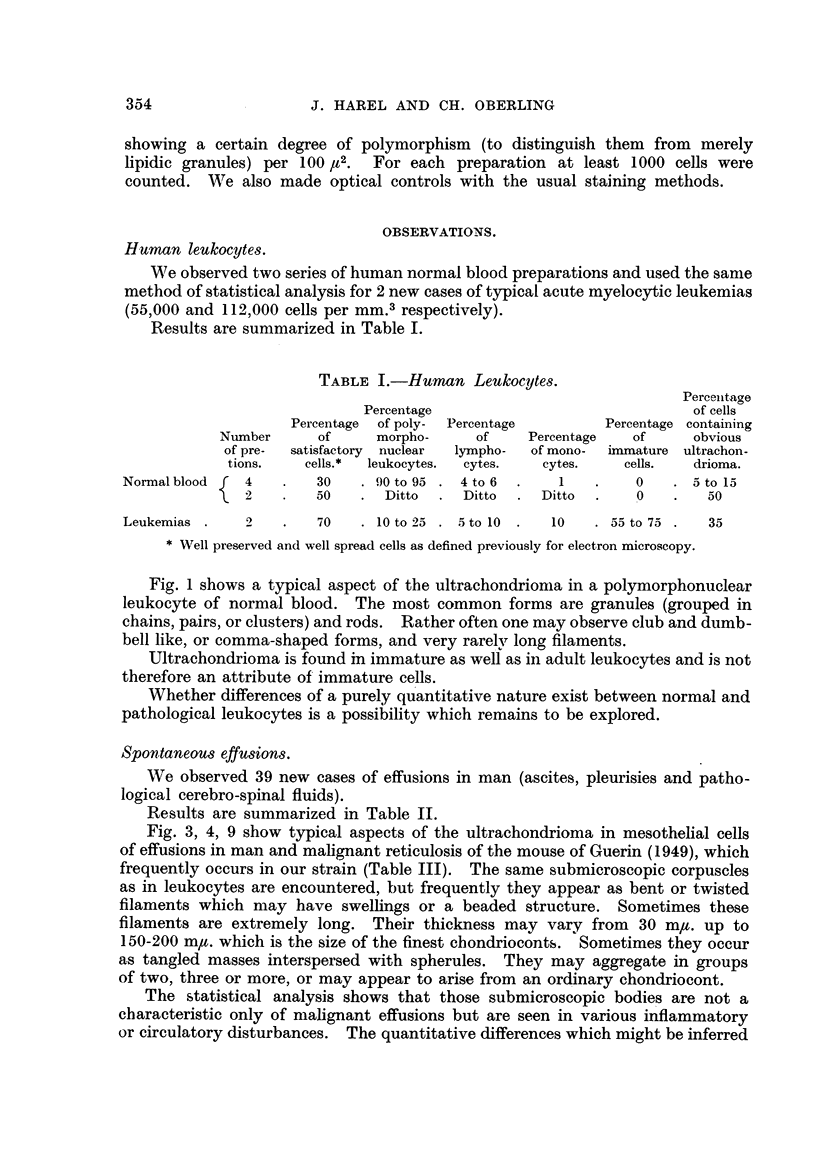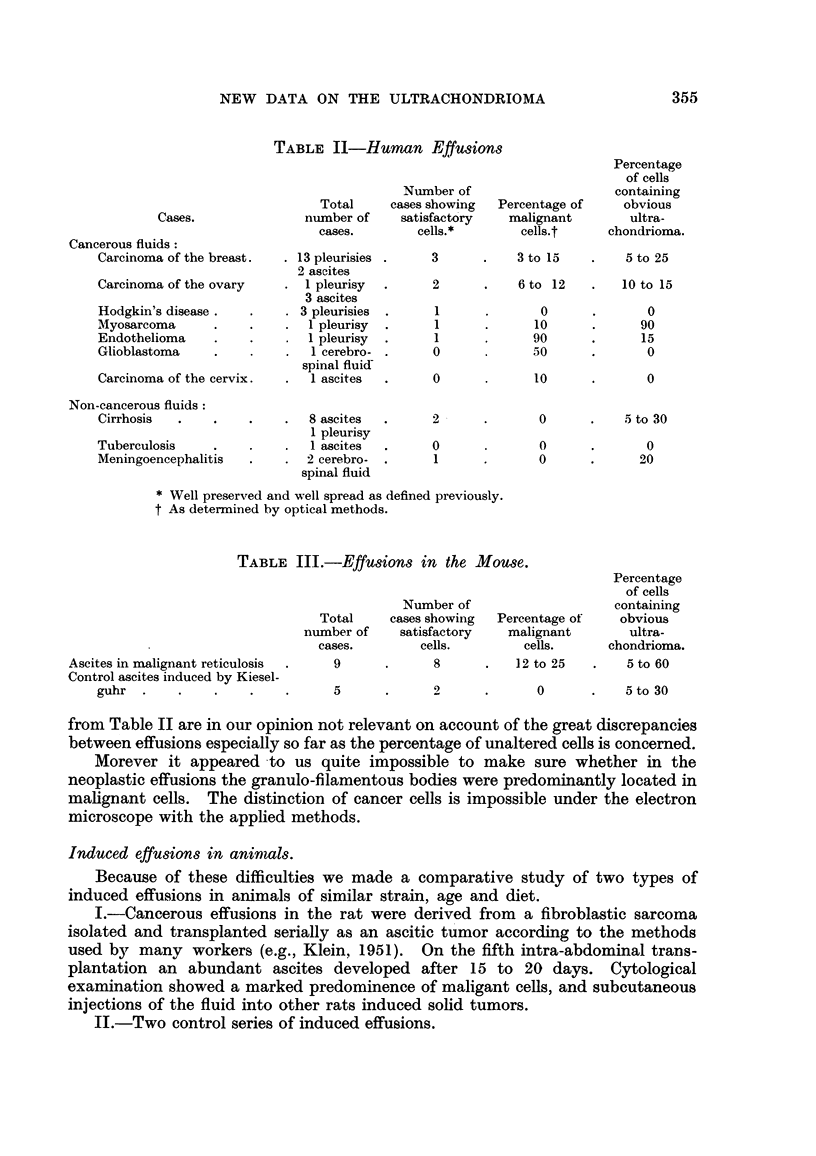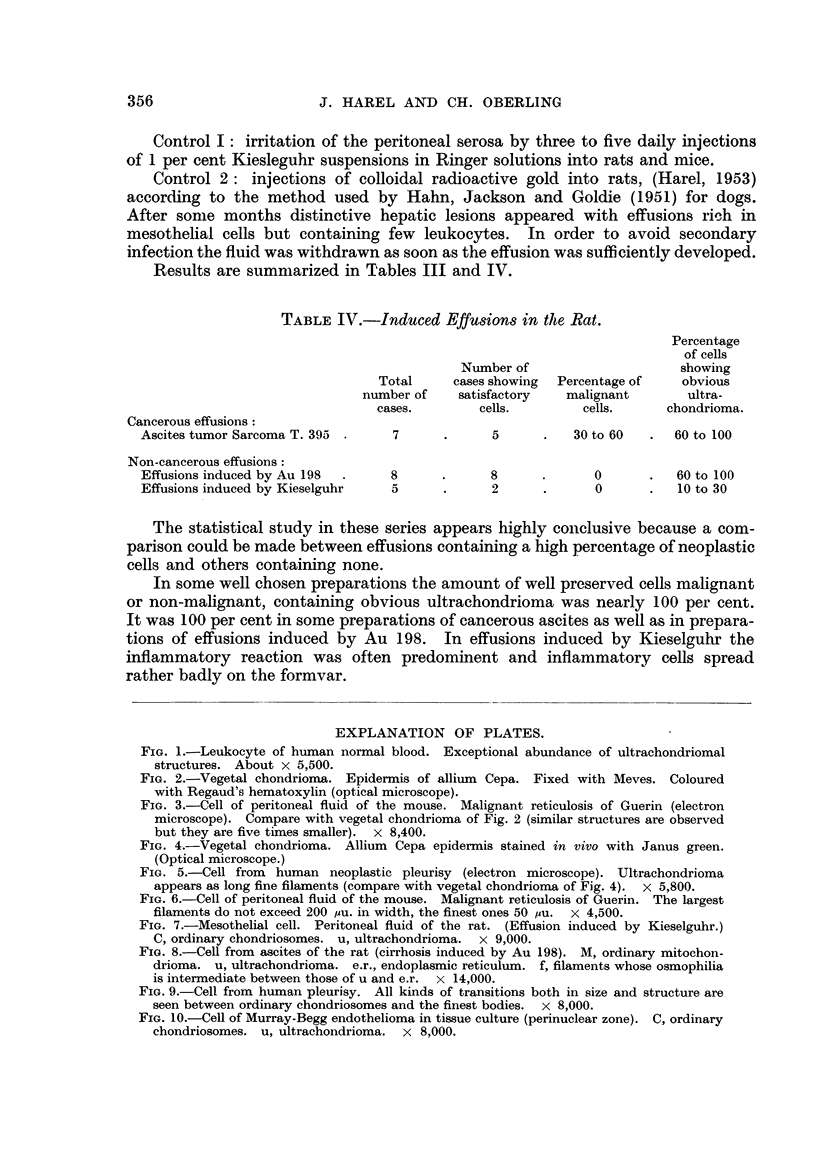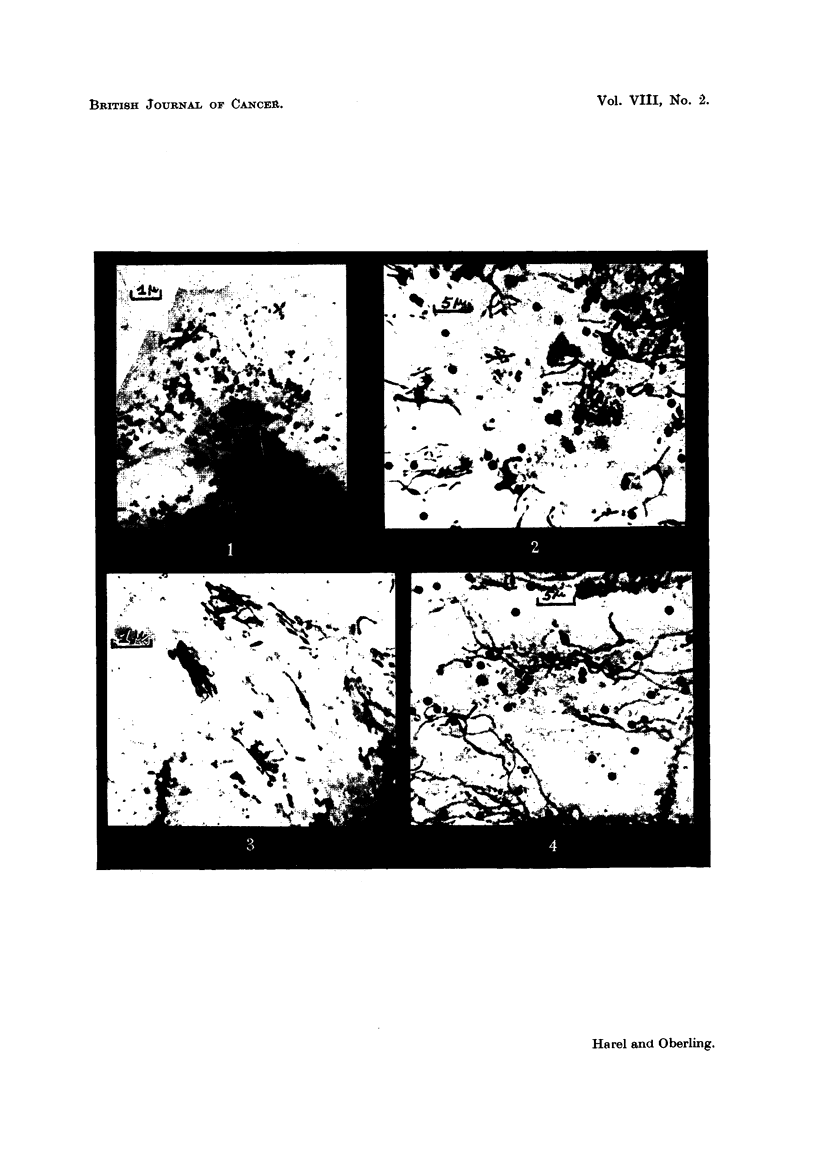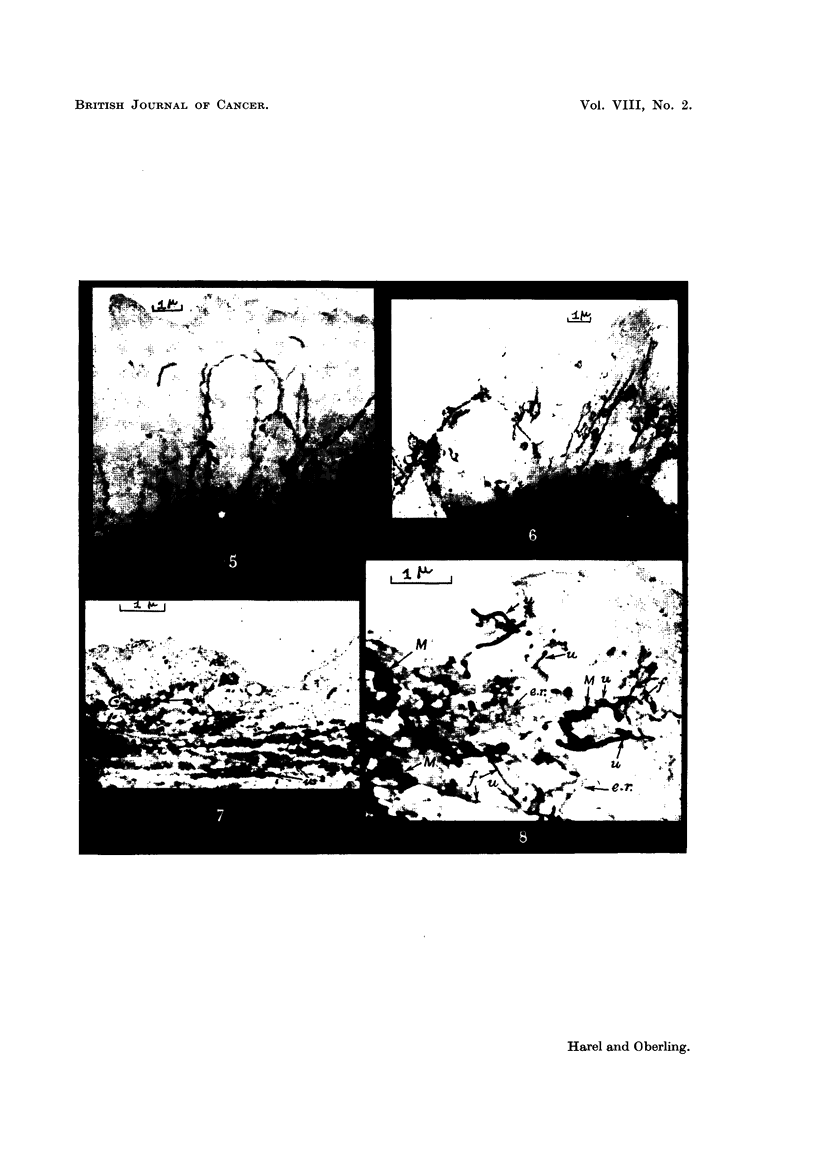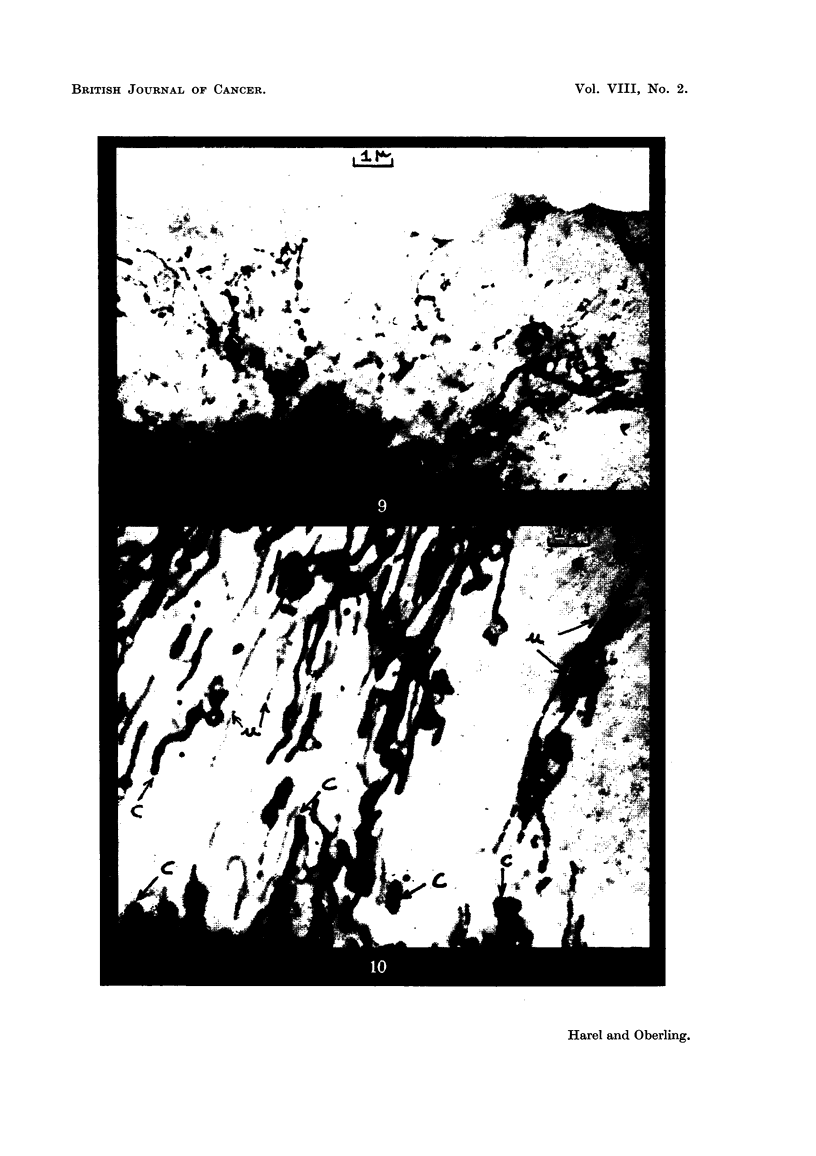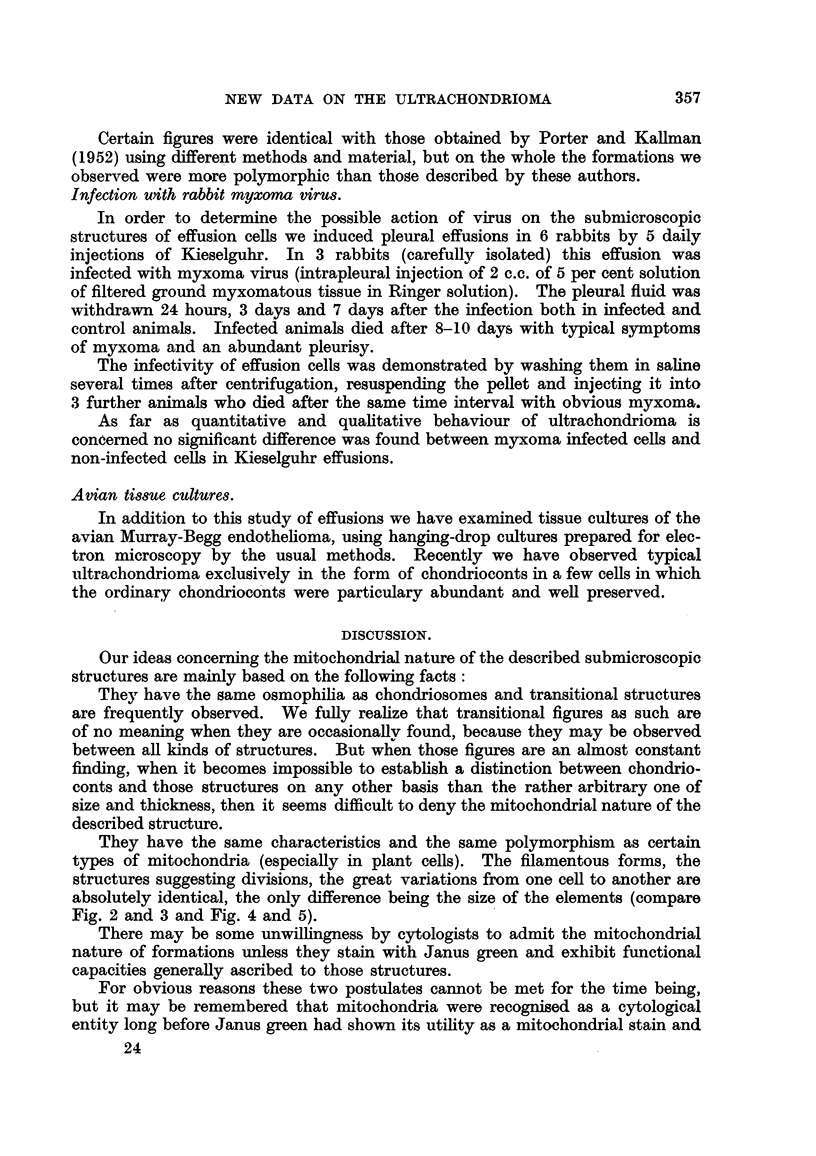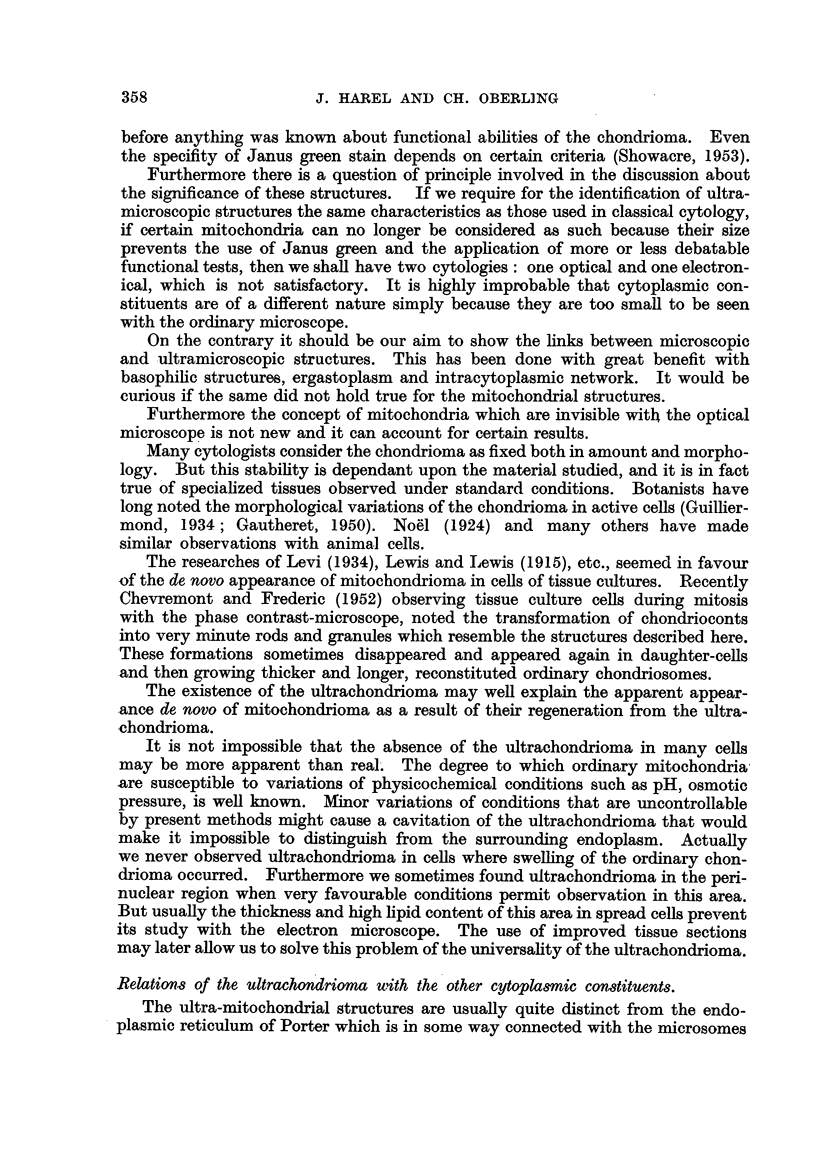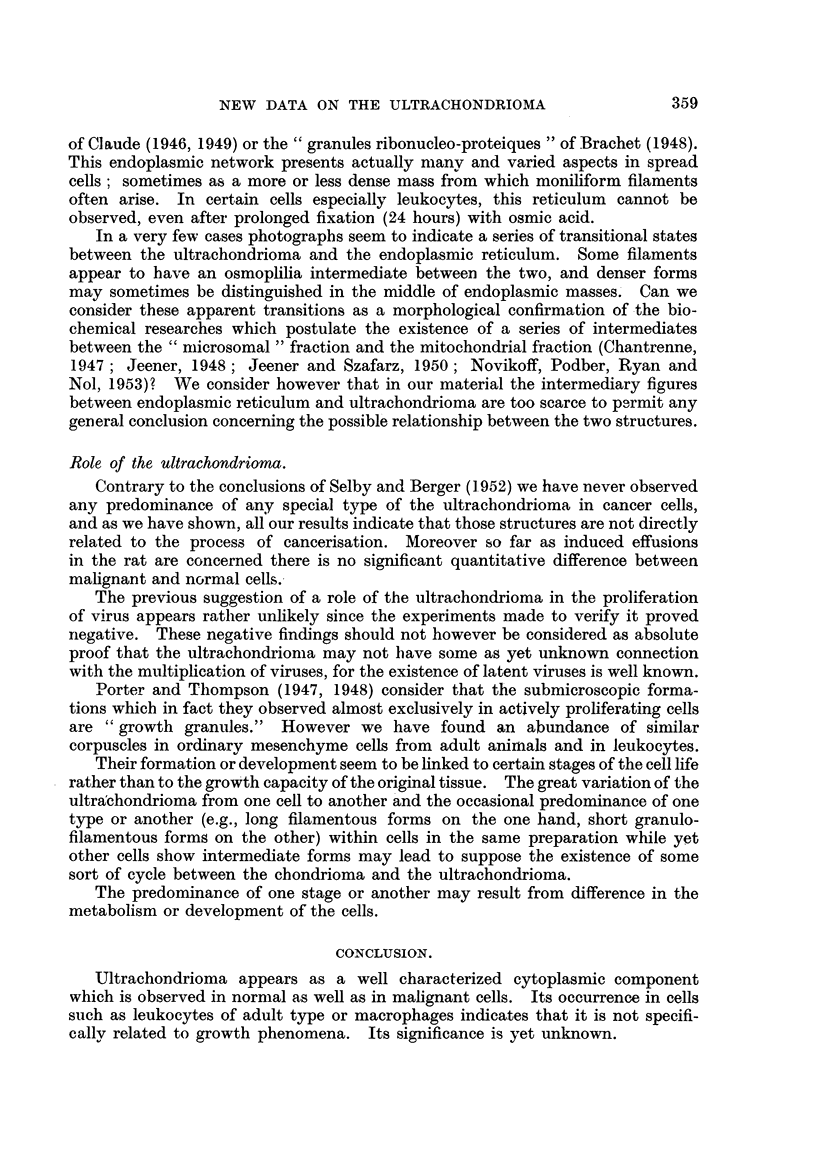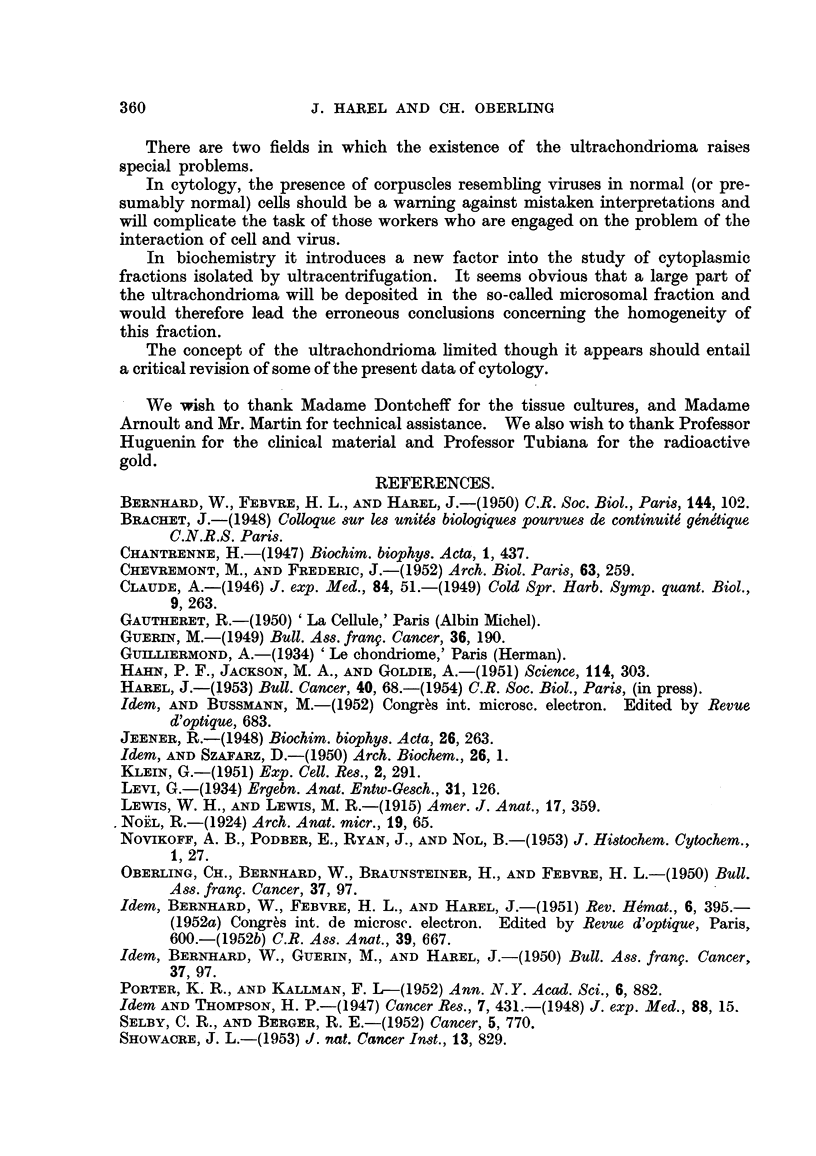# New Data on the Ultrachondrioma

**DOI:** 10.1038/bjc.1954.36

**Published:** 1954-06

**Authors:** J. Harel, Ch. Oberling

## Abstract

**Images:**


### NEW DATA ON THE ULTRACHONDRIOMA

J. HAREL AND CH. OBERLING.

*From the Institut de Recherches sur le Cancer Gustave Roussy, Villejuif (Seine) France.*

Received for publication March 13, 1954.

IN 1950 Oberling, Bernhard, Braunsteiner and Febvre noticed the presence of ultramicroscopic granules and rods in leukoblasts of human leukemias and discussed their possible relationship with corpuscular elements earlier observed by Porter and Thomson (1947 ; 1948) in cells of rat cysticercus sarcoma.

Further studies (Oberling, Bernhard, Guerin and Harel, 1950; Oberling, Bernhard, Febvre and Harel, 1951, 1952*a*, 1952*b*) revealed a great polymorphism of these elements, their evident similarity with mitochondrial structures and their frequent occurrence not only in neoplastic but also in normal cells. These results led Oberling and his co-workers to consider them as a submicroscopic chondrioma or ultrachondrioma.

Since then, Selby and Berger (1952) have described such formations in tissue cultures of human carcinomas while Porter and Kallman (1952) found them also in rat fibroblasts derived either from embryos or from sarcomas and called them " growth granules."

We have further studied these particles in the hope of elucidating their nature, and more especially their possible relationships with virus infections and with cancer.

### MATERIAL AND METHODS.

The methods used have already been described (Bernhard, Febvre and Harel, 1950 ; Harel and Bussmann, 1952). They take advantage of the spontaneous adhesion on plastic membranes of the free cells present in physiological fluids such as blood, cerebro-spinal fluid, peritoneal and pleural effusions.

In brief : glass slides coated with formvar are placed in contact with the fluid (with addition of heparin if necessary) in an incubator at 37° C. for 15 to 60 minutes.

The slides are then rinsed with Tyrode solution at 37°, fixed 10 minutes to several hours in acid osmic vapour and washed thoroughly in distilled water. The formvar is stripped from the slide, mounted on grids and allowed to dry at room temperature for at least 24 hours.

Photographs were taken with a Trüb-Taüber electron microscope at 60 kV. We adopted stringent criteria for purposes of comparison and considered only undamaged cells, that is to say cells having clear and well defined chondriosomes and with a satisfactory cytoplasmic transparency. As the appearance of a cell may vary from one area to another (because of differences in spreading, lipid constituents, etc.) we only considered the best portions. Counts were made in each cell of osmiophilic bodies less than 150 m$\mu$. in greatest diameter, in an area of 100 $\mu^2$. At least a few such bodies are to be found in every cell, but to consider them as true ultrachondrioma we adopted as a minimum 10 such particles showing a certain degree of polymorphism (to distinguish them from merely lipidic granules) per 100 $\mu^2$. For each preparation at least 1000 cells were counted. We also made optical controls with the usual staining methods.

### OBSERVATIONS.

*Human leukocytes.*

We observed two series of human normal blood preparations and used the same method of statistical analysis for 2 new cases of typical acute myelocytic leukemias (55,000 and 112,000 cells per mm.$^3$ respectively).

Results are summarized in Table I.

TABLE I.—*Human Leukocytes.*

| | Number of pre-tions. | Percentage of satisfactory cells.* | Percentage of poly-morpho-nuclear leukocytes. | Percentage of lympho-cytes. | Percentage of mono-cytes. | Percentage of immature cells. | Percentage of cells containing obvious ultrachon-drioma. |
|---|---|---|---|---|---|---|---|
| Normal blood | 4 | 30 | 90 to 95 | 4 to 6 | 1 | 0 | 5 to 15 |
| | 2 | 50 | Ditto | Ditto | Ditto | 0 | 50 |
| Leukemias | 2 | 70 | 10 to 25 | 5 to 10 | 10 | 55 to 75 | 35 |

* Well preserved and well spread cells as defined previously for electron microscopy.

Fig. 1 shows a typical aspect of the ultrachondrioma in a polymorphonuclear leukocyte of normal blood. The most common forms are granules (grouped in chains, pairs, or clusters) and rods. Rather often one may observe club and dumb-bell like, or comma-shaped forms, and very rarely long filaments.

Ultrachondrioma is found in immature as well as in adult leukocytes and is not therefore an attribute of immature cells.

Whether differences of a purely quantitative nature exist between normal and pathological leukocytes is a possibility which remains to be explored.

*Spontaneous effusions.*

We observed 39 new cases of effusions in man (ascites, pleurisies and pathological cerebro-spinal fluids).

Results are summarized in Table II.

Fig. 3, 4, 9 show typical aspects of the ultrachondrioma in mesothelial cells of effusions in man and malignant reticulosis of the mouse of Guerin (1949), which frequently occurs in our strain (Table III). The same submicroscopic corpuscles as in leukocytes are encountered, but frequently they appear as bent or twisted filaments which may have swellings or a beaded structure. Sometimes these filaments are extremely long. Their thickness may vary from 30 m$\mu$. up to 150-200 m$\mu$. which is the size of the finest chondrioconts. Sometimes they occur as tangled masses interspersed with spherules. They may aggregate in groups of two, three or more, or may appear to arise from an ordinary chondriocont.

The statistical analysis shows that those submicroscopic bodies are not a characteristic only of malignant effusions but are seen in various inflammatory or circulatory disturbances. The quantitative differences which might be inferred

TABLE II—*Human Effusions*

| Cases. | Total number of cases. | Number of cases showing satisfactory cells.* | Percentage of malignant cells.† | Percentage of cells containing obvious ultra-chondrioma. |
|---|---|---|---|---|
| Cancerous fluids : | | | | |
| Carcinoma of the breast | 13 pleurisies 2 ascites | 3 | 3 to 15 | 5 to 25 |
| Carcinoma of the ovary | 1 pleurisy 3 ascites | 2 | 6 to 12 | 10 to 15 |
| Hodgkin's disease | 3 pleurisies | 1 | 0 | 0 |
| Myosarcoma | 1 pleurisy | 1 | 10 | 90 |
| Endothelioma | 1 pleurisy | 1 | 90 | 15 |
| Glioblastoma | 1 cerebro-spinal fluid | 0 | 50 | 0 |
| Carcinoma of the cervix | 1 ascites | 0 | 10 | 0 |
| Non-cancerous fluids : | | | | |
| Cirrhosis | 8 ascites 1 pleurisy | 2 | 0 | 5 to 30 |
| Tuberculosis | 1 ascites | 0 | 0 | 0 |
| Meningoencephalitis | 2 cerebro-spinal fluid | 1 | 0 | 20 |

* Well preserved and well spread as defined previously.
† As determined by optical methods.

TABLE III.—*Effusions in the Mouse.*

| | Total number of cases. | Number of cases showing satisfactory cells. | Percentage of malignant cells. | Percentage of cells containing obvious ultra-chondrioma. |
|---|---|---|---|---|
| Ascites in malignant reticulosis | 9 | 8 | 12 to 25 | 5 to 60 |
| Control ascites induced by Kieselguhr | 5 | 2 | 0 | 5 to 30 |

from Table II are in our opinion not relevant on account of the great discrepancies between effusions especially so far as the percentage of unaltered cells is concerned.

Morever it appeared to us quite impossible to make sure whether in the neoplastic effusions the granulo-filamentous bodies were predominantly located in malignant cells. The distinction of cancer cells is impossible under the electron microscope with the applied methods.

*Induced effusions in animals.*

Because of these difficulties we made a comparative study of two types of induced effusions in animals of similar strain, age and diet.

I.—Cancerous effusions in the rat were derived from a fibroblastic sarcoma isolated and transplanted serially as an ascitic tumor according to the methods used by many workers (e.g., Klein, 1951). On the fifth intra-abdominal transplantation an abundant ascites developed after 15 to 20 days. Cytological examination showed a marked predominence of maligant cells, and subcutaneous injections of the fluid into other rats induced solid tumors.

II.—Two control series of induced effusions.

Control I : irritation of the peritoneal serosa by three to five daily injections of 1 per cent Kiesleguhr suspensions in Ringer solutions into rats and mice.

Control 2 : injections of colloidal radioactive gold into rats, (Harel, 1953) according to the method used by Hahn, Jackson and Goldie (1951) for dogs. After some months distinctive hepatic lesions appeared with effusions rich in mesothelial cells but containing few leukocytes. In order to avoid secondary infection the fluid was withdrawn as soon as the effusion was sufficiently developed.

Results are summarized in Tables III and IV.

TABLE IV.—*Induced Effusions in the Rat.*

| | Total number of cases. | Number of cases showing satisfactory cells. | Percentage of malignant cells. | Percentage of cells showing obvious ultra-chondrioma. |
|---|---|---|---|---|
| Cancerous effusions : | | | | |
| Ascites tumor Sarcoma T. 395 | 7 | 5 | 30 to 60 | 60 to 100 |
| Non-cancerous effusions : | | | | |
| Effusions induced by Au 198 | 8 | 8 | 0 | 60 to 100 |
| Effusions induced by Kieselguhr | 5 | 2 | 0 | 10 to 30 |

The statistical study in these series appears highly conclusive because a comparison could be made between effusions containing a high percentage of neoplastic cells and others containing none.

In some well chosen preparations the amount of well preserved cells malignant or non-malignant, containing obvious ultrachondrioma was nearly 100 per cent. It was 100 per cent in some preparations of cancerous ascites as well as in preparations of effusions induced by Au 198. In effusions induced by Kieselguhr the inflammatory reaction was often predominent and inflammatory cells spread rather badly on the formvar.

### EXPLANATION OF PLATES.

FIG. 1.—Leukocyte of human normal blood. Exceptional abundance of ultrachondriomal structures. About × 5,500.

FIG. 2.—Vegetal chondrioma. Epidermis of allium Cepa. Fixed with Meves. Coloured with Regaud's hematoxylin (optical microscope).

FIG. 3.—Cell of peritoneal fluid of the mouse. Malignant reticulosis of Guerin (electron microscope). Compare with vegetal chondrioma of Fig. 2 (similar structures are observed but they are five times smaller). × 8,400.

FIG. 4.—Vegetal chondrioma. Allium Cepa epidermis stained *in vivo* with Janus green. (Optical microscope.)

FIG. 5.—Cell from human neoplastic pleurisy (electron microscope). Ultrachondrioma appears as long fine filaments (compare with vegetal chondrioma of Fig. 4). × 5,800.

FIG. 6.—Cell of peritoneal fluid of the mouse. Malignant reticulosis of Guerin. The largest filaments do not exceed 200 $\mu$u. in width, the finest ones 50 $\mu$u. × 4,500.

FIG. 7.—Mesothelial cell. Peritoneal fluid of the rat. (Effusion induced by Kieselguhr.) C, ordinary chondriosomes. u, ultrachondrioma. × 9,000.

FIG. 8.—Cell from ascites of the rat (cirrhosis induced by Au 198). M, ordinary mitochondrioma. u, ultrachondrioma. e.r., endoplasmic reticulum. f, filaments whose osmophilia is intermediate between those of u and e.r. × 14,000.

FIG. 9.—Cell from human pleurisy. All kinds of transitions both in size and structure are seen between ordinary chondriosomes and the finest bodies. × 8,000.

FIG. 10.—Cell of Murray-Begg endothelioma in tissue culture (perinuclear zone). C, ordinary chondriosomes. u, ultrachondrioma. × 8,000.

1

2

3

4

1μ
1μ
6
5
1μ
u
M
u
M u
f
e.r.
C
u
u
M
u
f
u
e.r.
u
7
8

9

10

Harel and Oberling.

Certain figures were identical with those obtained by Porter and Kallman (1952) using different methods and material, but on the whole the formations we observed were more polymorphic than those described by these authors.

*Infection with rabbit myxoma virus.*

In order to determine the possible action of virus on the submicroscopic structures of effusion cells we induced pleural effusions in 6 rabbits by 5 daily injections of Kieselguhr. In 3 rabbits (carefully isolated) this effusion was infected with myxoma virus (intrapleural injection of 2 c.c. of 5 per cent solution of filtered ground myxomatous tissue in Ringer solution). The pleural fluid was withdrawn 24 hours, 3 days and 7 days after the infection both in infected and control animals. Infected animals died after 8–10 days with typical symptoms of myxoma and an abundant pleurisy.

The infectivity of effusion cells was demonstrated by washing them in saline several times after centrifugation, resuspending the pellet and injecting it into 3 further animals who died after the same time interval with obvious myxoma.

As far as quantitative and qualitative behaviour of ultrachondrioma is concerned no significant difference was found between myxoma infected cells and non-infected cells in Kieselguhr effusions.

*Avian tissue cultures.*

In addition to this study of effusions we have examined tissue cultures of the avian Murray-Begg endothelioma, using hanging-drop cultures prepared for electron microscopy by the usual methods. Recently we have observed typical ultrachondrioma exclusively in the form of chondrioconts in a few cells in which the ordinary chondrioconts were particulary abundant and well preserved.

### DISCUSSION.

Our ideas concerning the mitochondrial nature of the described submicroscopic structures are mainly based on the following facts :

They have the same osmophilia as chondriosomes and transitional structures are frequently observed. We fully realize that transitional figures as such are of no meaning when they are occasionally found, because they may be observed between all kinds of structures. But when those figures are an almost constant finding, when it becomes impossible to establish a distinction between chondrioconts and those structures on any other basis than the rather arbitrary one of size and thickness, then it seems difficult to deny the mitochondrial nature of the described structure.

They have the same characteristics and the same polymorphism as certain types of mitochondria (especially in plant cells). The filamentous forms, the structures suggesting divisions, the great variations from one cell to another are absolutely identical, the only difference being the size of the elements (compare Fig. 2 and 3 and Fig. 4 and 5).

There may be some unwillingness by cytologists to admit the mitochondrial nature of formations unless they stain with Janus green and exhibit functional capacities generally ascribed to those structures.

For obvious reasons these two postulates cannot be met for the time being, but it may be remembered that mitochondria were recognised as a cytological entity long before Janus green had shown its utility as a mitochondrial stain and

24

before anything was known about functional abilities of the chondrioma. Even the specifity of Janus green stain depends on certain criteria (Showacre, 1953).

Furthermore there is a question of principle involved in the discussion about the significance of these structures. If we require for the identification of ultramicroscopic structures the same characteristics as those used in classical cytology, if certain mitochondria can no longer be considered as such because their size prevents the use of Janus green and the application of more or less debatable functional tests, then we shall have two cytologies : one optical and one electronical, which is not satisfactory. It is highly improbable that cytoplasmic constituents are of a different nature simply because they are too small to be seen with the ordinary microscope.

On the contrary it should be our aim to show the links between microscopic and ultramicroscopic structures. This has been done with great benefit with basophilic structures, ergastoplasm and intracytoplasmic network. It would be curious if the same did not hold true for the mitochondrial structures.

Furthermore the concept of mitochondria which are invisible with the optical microscope is not new and it can account for certain results.

Many cytologists consider the chondrioma as fixed both in amount and morphology. But this stability is dependant upon the material studied, and it is in fact true of specialized tissues observed under standard conditions. Botanists have long noted the morphological variations of the chondrioma in active cells (Guilliermond, 1934 ; Gautheret, 1950). Noël (1924) and many others have made similar observations with animal cells.

The researches of Levi (1934), Lewis and Lewis (1915), etc., seemed in favour of the *de novo* appearance of mitochondrioma in cells of tissue cultures. Recently Chevremont and Frederic (1952) observing tissue culture cells during mitosis with the phase contrast-microscope, noted the transformation of chondrioconts into very minute rods and granules which resemble the structures described here. These formations sometimes disappeared and appeared again in daughter-cells and then growing thicker and longer, reconstituted ordinary chondriosomes.

The existence of the ultrachondrioma may well explain the apparent appearance *de novo* of mitochondrioma as a result of their regeneration from the ultrachondrioma.

It is not impossible that the absence of the ultrachondrioma in many cells may be more apparent than real. The degree to which ordinary mitochondria are susceptible to variations of physicochemical conditions such as pH, osmotic pressure, is well known. Minor variations of conditions that are uncontrollable by present methods might cause a cavitation of the ultrachondrioma that would make it impossible to distinguish from the surrounding endoplasm. Actually we never observed ultrachondrioma in cells where swelling of the ordinary chondrioma occurred. Furthermore we sometimes found ultrachondrioma in the perinuclear region when very favourable conditions permit observation in this area. But usually the thickness and high lipid content of this area in spread cells prevent its study with the electron microscope. The use of improved tissue sections may later allow us to solve this problem of the universality of the ultrachondrioma.

*Relations of the ultrachondrioma with the other cytoplasmic constituents.*

The ultra-mitochondrial structures are usually quite distinct from the endoplasmic reticulum of Porter which is in some way connected with the microsomes of Claude (1946, 1949) or the "granules ribonucleo-proteiques" of Brachet (1948). This endoplasmic network presents actually many and varied aspects in spread cells ; sometimes as a more or less dense mass from which moniliform filaments often arise. In certain cells especially leukocytes, this reticulum cannot be observed, even after prolonged fixation (24 hours) with osmic acid.

In a very few cases photographs seem to indicate a series of transitional states between the ultrachondrioma and the endoplasmic reticulum. Some filaments appear to have an osmoplilia intermediate between the two, and denser forms may sometimes be distinguished in the middle of endoplasmic masses. Can we consider these apparent transitions as a morphological confirmation of the biochemical researches which postulate the existence of a series of intermediates between the "microsomal" fraction and the mitochondrial fraction (Chantrenne, 1947 ; Jeener, 1948 ; Jeener and Szafarz, 1950 ; Novikoff, Podber, Ryan and Nol, 1953)? We consider however that in our material the intermediary figures between endoplasmic reticulum and ultrachondrioma are too scarce to permit any general conclusion concerning the possible relationship between the two structures.

*Role of the ultrachondrioma.*

Contrary to the conclusions of Selby and Berger (1952) we have never observed any predominance of any special type of the ultrachondrioma in cancer cells, and as we have shown, all our results indicate that those structures are not directly related to the process of cancerisation. Moreover so far as induced effusions in the rat are concerned there is no significant quantitative difference between malignant and normal cells.

The previous suggestion of a role of the ultrachondrioma in the proliferation of virus appears rather unlikely since the experiments made to verify it proved negative. These negative findings should not however be considered as absolute proof that the ultrachondrioma may not have some as yet unknown connection with the multiplication of viruses, for the existence of latent viruses is well known.

Porter and Thompson (1947, 1948) consider that the submicroscopic formations which in fact they observed almost exclusively in actively proliferating cells are "growth granules." However we have found an abundance of similar corpuscles in ordinary mesenchyme cells from adult animals and in leukocytes. Their formation or development seem to be linked to certain stages of the cell life rather than to the growth capacity of the original tissue. The great variation of the ultrachondrioma from one cell to another and the occasional predominance of one type or another (e.g., long filamentous forms on the one hand, short granulo-filamentous forms on the other) within cells in the same preparation while yet other cells show intermediate forms may lead to suppose the existence of some sort of cycle between the chondrioma and the ultrachondrioma.

The predominance of one stage or another may result from difference in the metabolism or development of the cells.

### CONCLUSION.

Ultrachondrioma appears as a well characterized cytoplasmic component which is observed in normal as well as in malignant cells. Its occurrence in cells such as leukocytes of adult type or macrophages indicates that it is not specifically related to growth phenomena. Its significance is yet unknown.

There are two fields in which the existence of the ultrachondrioma raises special problems.

In cytology, the presence of corpuscles resembling viruses in normal (or presumably normal) cells should be a warning against mistaken interpretations and will complicate the task of those workers who are engaged on the problem of the interaction of cell and virus.

In biochemistry it introduces a new factor into the study of cytoplasmic fractions isolated by ultracentrifugation. It seems obvious that a large part of the ultrachondrioma will be deposited in the so-called microsomal fraction and would therefore lead the erroneous conclusions concerning the homogeneity of this fraction.

The concept of the ultrachondrioma limited though it appears should entail a critical revision of some of the present data of cytology.

We wish to thank Madame Dontcheff for the tissue cultures, and Madame Arnoult and Mr. Martin for technical assistance. We also wish to thank Professor Huguenin for the clinical material and Professor Tubiana for the radioactive gold.

### REFERENCES.

Bernhard, W., Febvre, H. L., and Harel, J.—(1950) *C.R. Soc. Biol., Paris*, **144**, 102.
Brachet, J.—(1948) *Colloque sur les unités biologiques pourvues de continuité génétique C.N.R.S. Paris.*
Chantrenne, H.—(1947) *Biochim. biophys. Acta*, **1**, 437.
Chevremont, M., and Frederic, J.—(1952) *Arch. Biol. Paris*, **63**, 259.
Claude, A.—(1946) *J. exp. Med.*, **84**, 51.—(1949) *Cold Spr. Harb. Symp. quant. Biol.*, **9**, 263.
Gautheret, R.—(1950) 'La Cellule,' Paris (Albin Michel).
Guerin, M.—(1949) *Bull. Ass. franç. Cancer*, **36**, 190.
Guilliermond, A.—(1934) 'Le chondriome,' Paris (Herman).
Hahn, P. F., Jackson, M. A., and Goldie, A.—(1951) *Science*, **114**, 303.
Harel, J.—(1953) *Bull. Cancer*, **40**, 68.—(1954) *C.R. Soc. Biol., Paris*, (in press).
*Idem*, and Bussmann, M.—(1952) Congrès int. microsc. electron. Edited by *Revue d'optique*, 683.
Jeener, R.—(1948) *Biochim. biophys. Acta*, **26**, 263.
*Idem*, and Szafarz, D.—(1950) *Arch. Biochem.*, **26**, 1.
Klein, G.—(1951) *Exp. Cell. Res.*, **2**, 291.
Levi, G.—(1934) *Ergebn. Anat. Entw-Gesch.*, **31**, 126.
Lewis, W. H., and Lewis, M. R.—(1915) *Amer. J. Anat.*, **17**, 359.
Noël, R.—(1924) *Arch. Anat. micr.*, **19**, 65.
Novikoff, A. B., Podber, E., Ryan, J., and Nol, B.—(1953) *J. Histochem. Cytochem.*, **1**, 27.
Oberling, Ch., Bernhard, W., Braunsteiner, H., and Febvre, H. L.—(1950) *Bull. Ass. franç. Cancer*, **37**, 97.
*Idem*, Bernhard, W., Febvre, H. L., and Harel, J.—(1951) *Rev. Hémat.*, **6**, 395.—(1952*a*) Congrès int. de microsc. electron. Edited by *Revue d'optique*, Paris, 600.—(1952*b*) *C.R. Ass. Anat.*, **39**, 667.
*Idem*, Bernhard, W., Guerin, M., and Harel, J.—(1950) *Bull. Ass. franç. Cancer*, **37**, 97.
Porter, K. R., and Kallman, F. L—(1952) *Ann. N.Y. Acad. Sci.*, **6**, 882.
*Idem* and Thompson, H. P.—(1947) *Cancer Res.*, **7**, 431.—(1948) *J. exp. Med.*, **88**, 15.
Selby, C. R., and Berger, R. E.—(1952) *Cancer*, **5**, 770.
Showacre, J. L.—(1953) *J. nat. Cancer Inst.*, **13**, 829.